# Condyle dislocation following mandibular reconstruction using a fibula free flap: complication cases

**DOI:** 10.1186/s40902-019-0197-1

**Published:** 2019-04-01

**Authors:** Sang-Hoon Kang, Sanghoon Lee, Woong Nam

**Affiliations:** 1 Department of Oral and Maxillofacial Surgery, Yonsei University, College of Dentistry, 50 Yonsei-ro, Seodaemun-gu, Seoul, 03722 Republic of Korea; 2 Department of Oral and Maxillofacial Surgery, National Health Insurance Service Ilsan Hospital, 100 Ilsan-ro, Ilsan-donggu, Goyang, Gyeonggi-do 10444 Republic of Korea

**Keywords:** Mandibular reconstruction, Complication, Condyle dislocation, Fibular free flap

## Abstract

**Background:**

Condylar dislocation can arise as a complication in patients who required mandibular and/or condylar reconstruction and were operated on with fibula free flap (FFF) using surgical guides designed using simulation surgery. Surgeons should be aware of the complications in these present cases when planning and performing reconstructions as well as predicting prognoses.

**Cases presentation:**

Two cases showed condylar dislocation in mandibular reconstruction using a FFF fixed with a reconstruction plate. Three cases showed condylar dislocation in mandibular reconstruction using a fibula free flap fixed with a mini-plate.

**Conclusion:**

Despite the lack of clinical symptoms in these cases following mandibular reconstruction using an FFF, the mandibular condyle was severely displaced away from the glenoid fossa. A surgeon must have sufficient time to consider the use of a long flap with thickness similar to that of the mandible, ways to minimize span and bending, and methods of fixation. The patient, moreover, should be educated on condylar dislocation. Customized CAD/CAM-prototyped temporomandibular condyle-connected plates may be a good alternative even if virtual simulation surgery is to be performed before surgery. These considerations may help reduce the incidence of complications after mandibular reconstruction.

## Background

The fibula free flap (FFF) is one of the most widely used flaps for the reconstruction of mandibular defects caused by trauma or lesions such as tumors [1, 2]. The FFF has several advantages including sufficient bone length for mandibular reconstruction, a high survival rate, and performability at the time of skin grafting [3–5].

Owing to recent advances in computer technology, it is now possible to perform virtual surgical planning (VSP) before surgery. It is also possible to convert information regarding the location of the osteotomy line and bone movements from preoperative mandibular reconstruction simulation and planning into stereolithography (STL) data. This can be done with computer-aided design/computer-aided manufacturing (CAD/CAM) for use in the development and deployment of surgical guides [6–9].

By using VSP and VSP-based CAD/CAM surgical guides, the original jaw bone shape can be reconstructed more accurately than when using pre-surgical computed tomography (CT) data of the jawbone and fibula [7, 10]. The guides reduce the size of bone segments in fibular bone grafting and enable more sophisticated reconstruction [3]. In addition, mandibular reconstruction using fibula cutting surgical guides can reduce surgical time [11]. However, cutting guides must be designed based on data obtained from virtual surgical planning and produced with a three-dimensional (3D)-printer that uses biocompatible materials.

Furthermore, bone fixation plates used in surgery are bent using a rapid prototyping (RP) mandibular model reconstructed as a fibula in virtual surgical planning to make the localization and fixation of bone segments easier [12].

Existing methods of mandibular reconstruction using the fibula have limitations. An examiner must perform plating, design a free flap himself in the operation room, and resect the jawbone and fibula by visually measuring them. However, even if these limitations are overcome by VSP and surgical guides, it is difficult to predict the level of mandibular functioning and long-term prognoses after surgery.

If the mandibular condyle in the temporomandibular joint (TMJ) must be completely removed, it must eventually be reconstructed. Methods of connecting customized condylar prostheses in patients treated with free flaps have also been reported [13, 14].

This report showed the cases of condylar dislocation in patients who required mandibular and/or condylar reconstruction, had a simulation surgery using VSP before surgery, and were operated on with FFF using surgical guides. Surgeons may refer to these present cases when planning and performing reconstructions as well as predicting prognoses. Surgeons should also be aware of the complications following mandibular reconstruction presented here.

## Cases presentation

### Condylar dislocation in mandibular reconstruction using a fibula free flap fixed with a reconstruction plate

#### Case 1

**Fig. 1:**
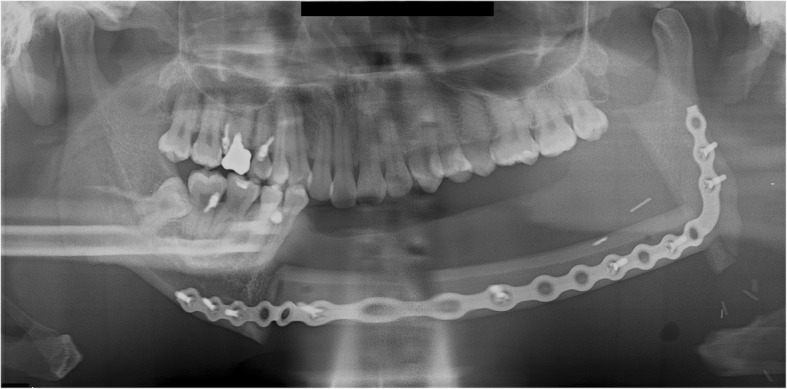
A panoramic radiograph obtained immediately after reconstruction using FFF following left mandibular resection

A 36-year-old male patient was diagnosed with a malignant nerve sheath tumor (MNST) of the left mandible and underwent mandibular resection. A VSP simulation surgery was then performed and CAD/CAM surgical guides were fabricated using VSP data. The mandibular bone from the right mandibular canine to the left ascending ramus was removed and reconstructed with an FFF, which was fixed with a reconstruction metal plate. The fibula was bent once and fixed on the right mandible and the left ascending ramus with a reconstruction plate (Fig. 1). Postoperative sag of left condylar segment was found in the panoramic view after surgery.

**Fig. 2:**
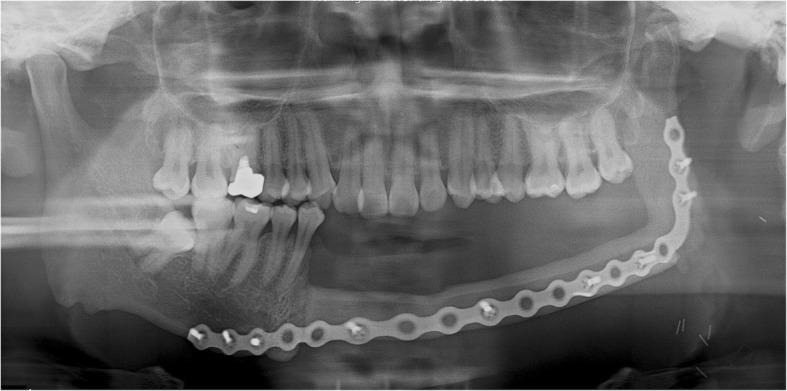
A panoramic radiograph obtained 8 months after reconstruction using FFF following left mandibular resection. The left mandibular condyle deviated from the glenoid fossa and became displaced in the anterior direction

Trismus was gradually relieved after surgery, with no pain around the TMJ during mandibular function. No open bite was observed in the right posterior mandible during mastication. During the postoperative follow-up, panoramic radiographs obtained 8 months after surgery showed anterior dislocation of the left condyle with a reduction of the interocclusal distance. The patient neither complained nor showed any signs of trismus or pain in the TMJ (Fig. 2).

#### Case 2

**Fig. 3:**
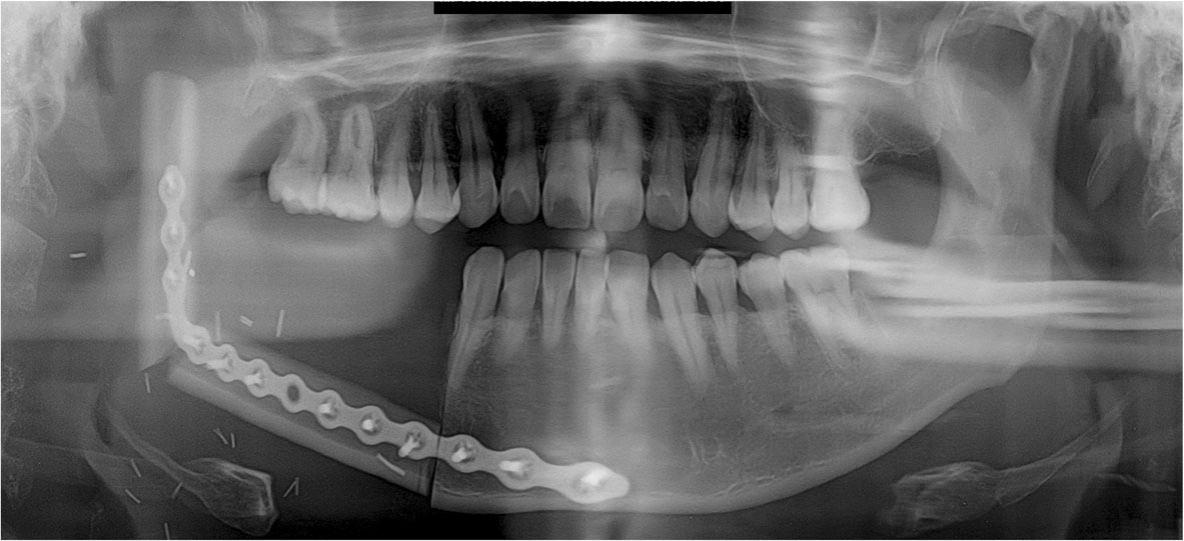
A panoramic radiograph obtained immediately after reconstruction using FFF following right mandibular resection

A 43-year-old male patient was diagnosed with squamous cell carcinoma (SCC stage IVa) of the right mandible and underwent neck dissection including mandibular and wide resections. A VSP simulation surgery was then performed and CAD/CAM surgical guides were fabricated using VSP data. The portion of the mandibular bone from the right mandibular premolar tooth to the right condyle and TMJ was removed and reconstructed with an FFF. The FFF was fixed with a reconstruction metal plate. The fibula was bent once, connected posteriorly to the right mandibular canine, and located on the glenoid fossa with a reconstruction plate (Fig. 3).

**Fig. 4:**
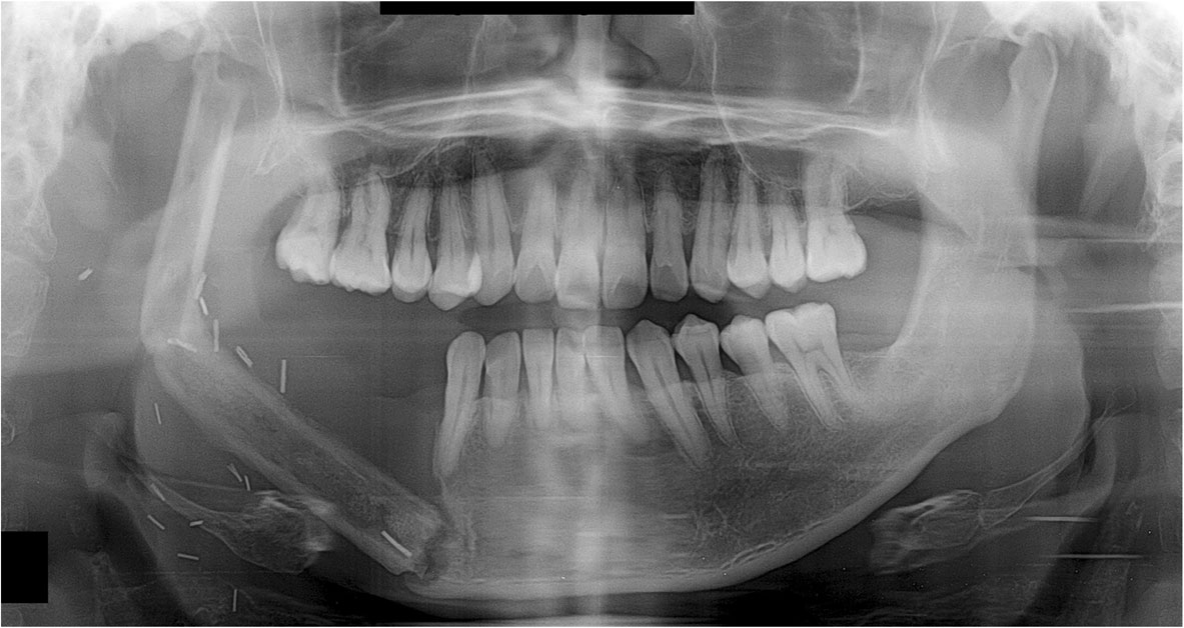
A panoramic radiograph obtained 2 years and 1 month after reconstruction using FFF following right mandibular resection. The fibular segment corresponding to the right mandibular condyle deviated from the glenoid fossa and became severely displaced. The fibular segment at the connection with the mandible is also severely displaced

Trismus was gradually relieved after surgery and there was no pain around the TMJ during mandibular function. No open bite was observed from the right mandibular canine to the left posterior region during mastication. During the postoperative follow-up, panoramic radiographs obtained 2 years and 1 month after surgery showed anterior dislocation of a segment of the fibula corresponding to the right condyle. The right posterior interocclusal space became narrower. The patient did not have trismus or pain in the TMJ (Fig. 4). The connection between the fibular segments 2 years later was found to be in a position similar to that observed immediately after the surgery. However, the connection between the anterior parts of the fibular segments and the mandible were severely displaced after surgery. Following radiation treatment after surgery, the metal plate became exposed and was subsequently removed. This sign may be resulted from the complication of reconstruction plate.

### Condylar dislocation in mandibular reconstruction using a fibula free flap fixed with a mini-plate

#### Case 3

**Fig. 5:**
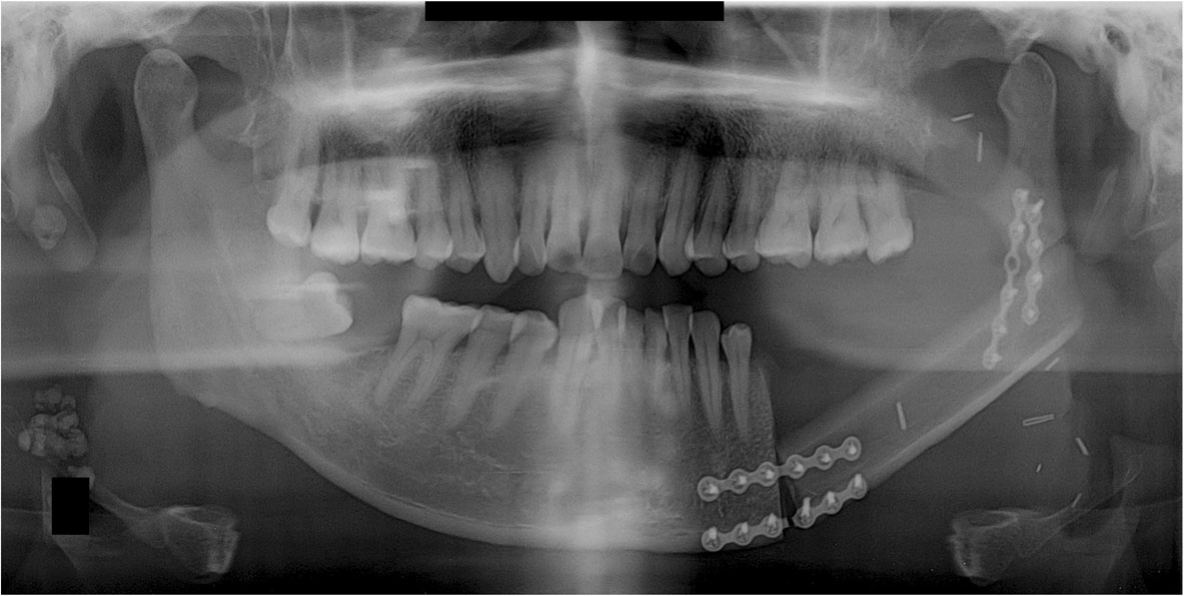
A panoramic radiograph obtained immediately after reconstruction using FFF following left mandibular resection. Of the miniplates at the connection between the remaining mandible and the fibular segments, those located at the bottom failed

A 47-year-old male patient was diagnosed with squamous cell carcinoma (SCC stage IVa) of the left mandible and underwent neck dissection including mandibular wide resections. A VSP simulation surgery was then performed and CAD/CAM surgical guides were fabricated using VSP data prior to the main surgery. The portion of the mandible from the left mandibular molar to the left ascending ramus was removed and reconstructed with an FFF. The FFF was fixed with miniplates using two plates per connection point. Fibulae were connected to the ascending ramus of the left mandible, their anterior portions being connected posteriorly to the mandibular premolar region. The fibulae were then fixed with miniplates (Fig. 5).

**Fig. 6:**
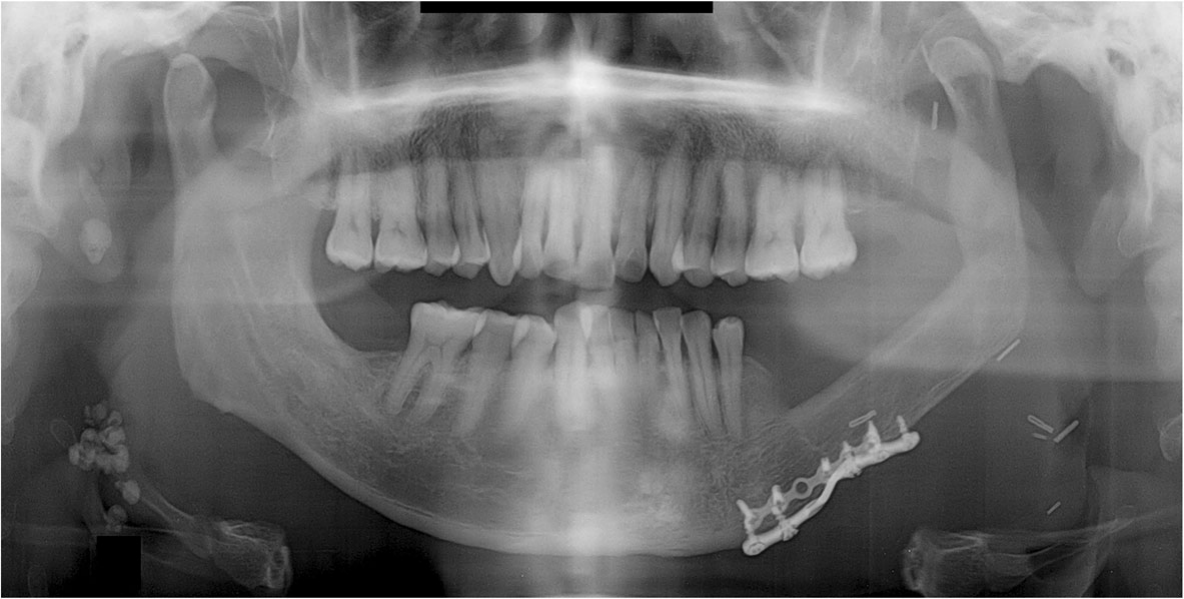
A panoramic radiograph obtained 3 years 1 month after reconstruction using FFF following left mandibular resection. The left mandibular condyle deviated from the glenoid fossa and became displaced in the anterior direction

Trismus was gradually relieved after surgery, with no pain around the TMJ during mandibular function. No open bite was observed from the right mandibular incisor to the right posterior region during mastication. The miniplates at the connection between the fibula and the mandible had failed and required reinforcement through additional fixation. During the postoperative follow-up, panoramic radiographs obtained 3 years and 1 month after surgery showed anterior dislocation of the left condyle. The left posterior interocclusal space had become narrower. The patient did not have trismus or pain in the TMJ (Fig. 6). The connection between the fibula segment and ascending ramus was found to be in a position similar to that observed immediately after surgery except for displacement between the anterior part of the fibula segment and the mandible. The miniplates at the connection between the fibula and the mandible had failed with widening of the inferior border. This sign may mean that this complication resulted from inappropriate height of ramus from gonial angle area to the condyle portion.

#### Case 4

**Fig. 7:**
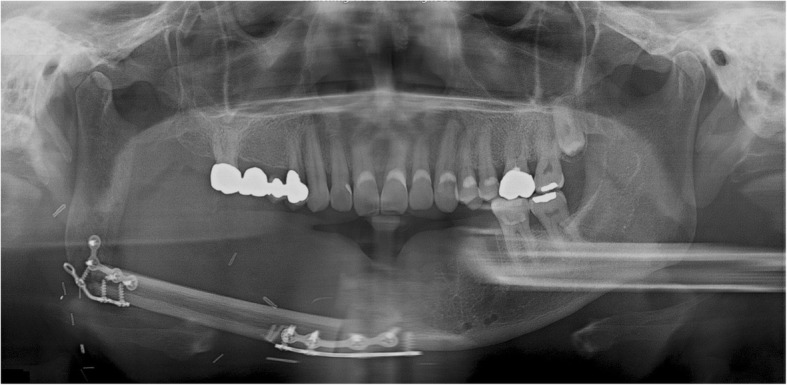
A panoramic radiograph obtained immediately after reconstruction using FFF following right mandibular resection. Of the miniplates at the connection between the remaining mandible and the fibular segments, those located at the bottom failed

A 61-year-old female patient diagnosed with squamous cell carcinoma (SCC stage III) of the right mandible underwent neck dissection including mandibular and side resections. A VSP simulation surgery was then performed and CAD/CAM surgical guides were fabricated using VSP data. The mandible from the right mandibular incisor to the right ascending ramus was removed and reconstructed with FFF. The FFF was fixed with miniplates, using two plates per connection point. The fibulae were connected to the ascending ramus of the right mandible, and their anterior portions were connected to the right mandibular parasymphysis and fixed with miniplates (Fig. 7).

**Fig. 8:**
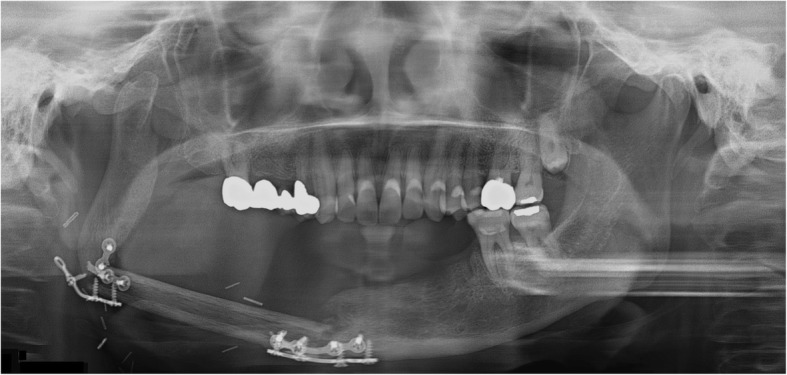
A panoramic radiograph obtained 1 year 3 months after reconstruction using FFF following right mandibular resection. The right mandibular condyle deviated from the glenoid fossa and became displaced in the anterior direction

Trismus was gradually relieved after surgery, with no pain around the TMJ during mandibular function. No open bite was observed on the left mandibular molar area during mastication. During the postoperative follow-up, panoramic radiographs obtained 1 year and 3 months after surgery showed anterior dislocation of the right condyle. The miniplates at the connection between the fibula and the right mandibular ascending ramus had failed. The connection between the fibular segment and the anterior mandibular region was found to be in a similar state as that observed immediately after surgery. The interocclusal space had not narrowed in the right posterior region, and the right mandibular condyle had become dislocated in the anterior direction. This apparently resulted from a failure to maintain the connection between the fibular segment and ascending ramus, leading to severe displacement (Fig. 8). The miniplates at bottom gonial angle area had failed the connection between the fibula and the right mandibular ascending ramus. The bottom gonial plate has been fixed only with one screw on the distal segment of the fibula. And finally, superior miniplate was broken. This sign means that lack of stability may cause the stress-related fatigue fracture of the plate and displace condylar segment.

#### Case 5

**Fig. 9:**
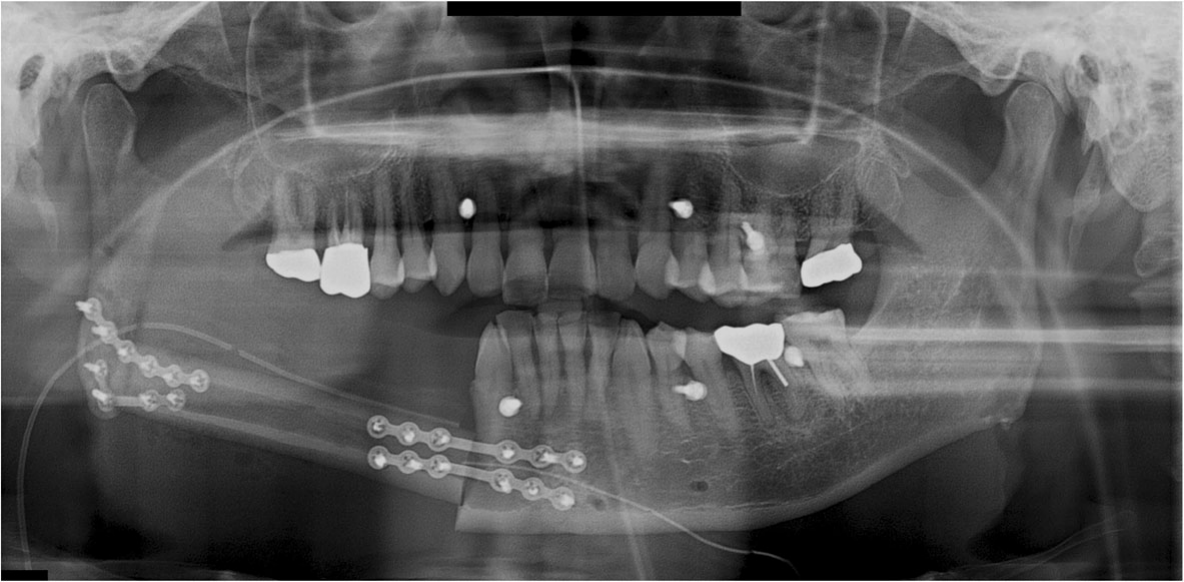
A panoramic radiograph obtained immediately after reconstruction using FFF following right mandibular resection

A 70-year-old male patient was diagnosed with squamous cell carcinoma (SCC stage IVa) of the right mandible and underwent neck dissection including mandibular and side resections. A VSP simulation surgery was then performed and CAD/CAM surgical guides were fabricated using VSP data. The mandibular bone from the right mandibular premolar to the right ascending ramus was removed and reconstructed with FFF fixed with miniplates, using two plates per connection point. The fibulae were connected to the ascending ramus of the right mandible, their anterior portions being connected posteriorly to the right mandibular incisors and fixed with miniplates (Fig. 9).

**Fig. 10:**
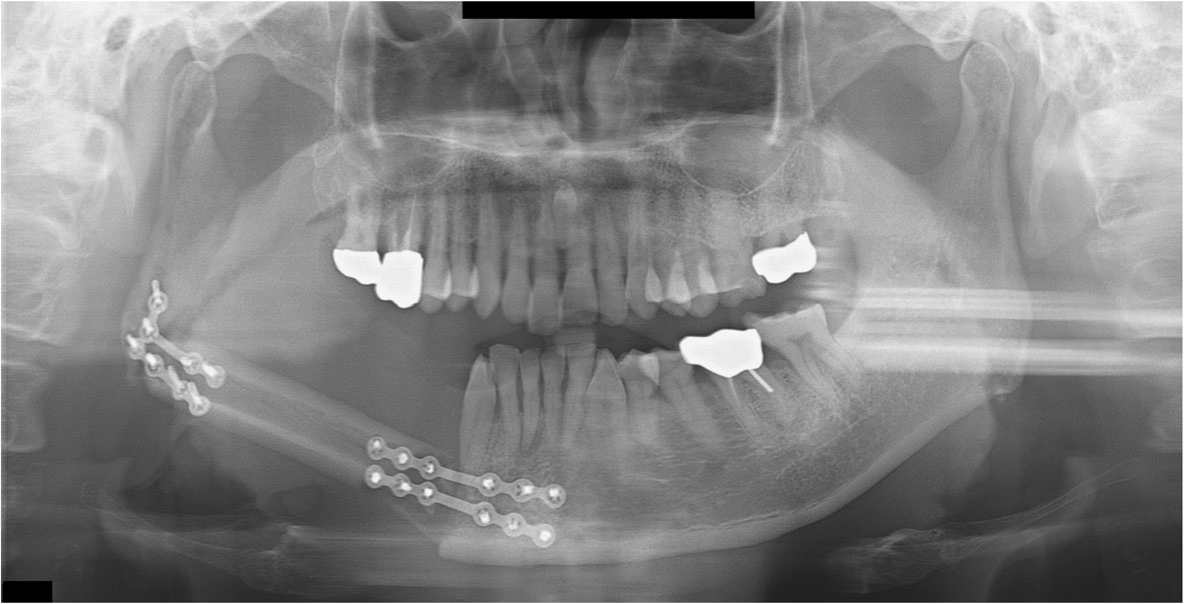
A panoramic radiograph obtained 5 months after reconstruction using FFF following right mandibular resection. The right mandibular condyle deviated from the glenoid fossa and became displaced in the anterior direction

Trismus was gradually relieved after surgery, with no pain around the TMJ during mandibular function. No open bite was observed from the anterior mandible to the left molar region during mastication. Panoramic radiographs obtained 5 months after surgery during postoperative follow-up showed anterior dislocation of the right condyle. The connection between the fibular segment and the anterior mandible was bent in the posterior direction relative to its original position immediately after surgery. The connection between the fibular segment and ascending ramus could not be maintained and became bent relative to the angle of the mandible immediately after surgery. Although the interocclusal space in the right posterior region had not narrowed, the fibular segments were bent inward, while the right mandibular condyle was displaced in the anterior direction (Fig. 10).

The metal plates at the connection between the fibulae and the right mandibular ascending ramus neither failed nor became exposed. Displacement of fibular proximal segment may be associated with the fibular bone destruction of junction area. This may cause the displacement of condylar portion.

### Three-dimensional simulations of mandibular reconstructions with fibula grafts

The DICOM (Digital Imaging and Communications in Medicine) files of the mandibular and fibular CT images were imported into the Mimics, version 14.0 software (Materialise, Leuven, Belgium). Then, three-dimensional (3D) images of the mandible and fibula were reconstructed. Thus, the simulated mandible was cut on the pathologic region according to the plan of operation. The 3D fibula graft was positioned at the sectioned mandibular region for mandibular reconstruction method. This simulation of the mandible reconstructed with the fibula was repeated and finally confirmed by operator. We then used stereolithographic (STL) data and a 3D printer (ProJet 360, 3D Systems, Inc., Rock Hill, SC) to manufacture rapid prototype (RP) models of reconstructed mandibles with FFF.

We manufactured a fibula cutting guide to facilitate cutting the fibula according to the surgical simulation. First, we designed the fibula cutting guide in the Mimics software. We moved the fibula bone fragments that were used to reconstruct the mandible to their original positions in the intact fibula bone. We rendered planes that would guide cutting, based on the cross sections of the fibula fragments. We used the STL data of the designed fibula cutting guide to manufacture the fibula cutting guide with the 3D printer (ProJet 3500 HDMax 3D Printer, 3D Systems, Inc., Rock Hill, SC). To facilitate placing the fibula segments into the mandible, we designed a fibula bending guide for each mandible in a reconstruction simulation. Based on the STL data of this design, the fibula bending guide was manufactured with the above same 3D printer.

## Discussion

In all cases, reconstruction of the mandible including the condylar region using fibulae during mandibular resection experienced condylar dislocation (Table 1). The inclusion of condylar resection in mandibular resection makes it difficult to determine whether to leave the condyle or not. When there is a large lesion, the mandibular condyle and TMJ are resected to remove the lesion. In this case, a reconstruction metal plate with the mandibular condyle head part can be used for reconstruction. However, the metal plate may become displaced into the cranium, and if the meniscus of the TMJ is removed prior to removal of the lesion, a basilar impression of the prosthesis in the form of a condyle may result. In such cases, the condyle may become dislocated, as observed in the present study [15]. It is advisable to perform mandibular reconstruction again as quickly as possible in such complication cases. All patients in the current cases underwent mandibular resection and reconstruction using FFF at the same time. One out of the five patients underwent condylar resection.

**Table 1:** Characteristics of five reported cases showing condyle dislocation following mandibular reconstruction using a fibula free flap

Case	Age/sex	Pathology	Region	Fixation method	VSP CAD/CAM surgical guide	Mandibulectomy and reconstruction area	Estimated cause of condylar displacement
1	36 M	MNST	Mandible, Lt.	Reconstruction plate	Fibula cutting/bending guide, RP	Right mandibular canine—left ascending ramus	Immediately postoperative sag of left condylar segment
2	43 M	SCC, stage IVa	Mandible, Rt.	Reconstruction plate	Fibula cutting/bending guide, RP	Right mandibular premolar—right condyle	Complication of reconstruction plate
3	47 M	SCC, stage IVa	Mandible, Lt.	Miniplate	Fibula cutting/bending guide, RP	Left mandibular molar—left ascending ramus	Inappropriate height of ramus
4	61 F	SCC, stage III	Mandible, Rt.	Miniplate	Fibula cutting/bending guide, RP	Right mandibular incisor—right ascending ramus	Instability and the stress-related fatigue plate fracture
5	70 M	SCC, stage IVa	Mandible, Rt.	Miniplate	Fibula cutting/bending guide, RP	Right mandibular premolar—right ascending ramus	Fibular bone destruction of junction area

*MNST* malignant nerve sheath tumor, *SCC* squamous cell carcinoma, *FFF* fibula free flap, *VSP* virtual surgical planning, *RP* rapid prototyping model

With the advent of VSP, which allows surgeons to perform a 3D simulation surgery prior to an actual surgery, surgical outcomes of mandibular reconstruction using a fibula have improved [16, 17]. It has been reported that serially performing mandibular resection followed by reconstruction using FFF incurs no increased risk of complications and yields functional outcomes similar to those of existing routine surgeries [16]. A 3D simulation surgery was performed for all patients prior to the actual surgery in this study. Angiographs of the peroneal arteries around the fibula were assessed, FFF was formed, and reconstruction was performed. None of the patients had a failed vascular anastomosis or problems with the FFF. For this reason, we cannot conclude that the dislocation of the fibular segment corresponding to the condylar region following reconstruction caused the failed bone healing of bony segments. Additionally, since a simulation surgery had been performed and surgical guides were used during the actual surgery, we cannot say that the fibular segment corresponding to the condylar region became displaced due to errors that arose during the osteotomy and positioning of the fibular segments during the surgery.

When shaving and connecting the resected bone segments of the FFF according to the surgical guides, reconstruction metal plates or miniplates can be used at every connection between each bone segment. In a biomechanical study, two 2.0-mm miniplates, a single 2.3-mm plate, and a single 2.7-mm plate were used at the fibular resection sites in three different groups, respectively. In this study, the 2.3-mm and 2.7-mm metal plates withstood greater forces than the two miniplates [18]. Again, in the current cases, condylar displacement was observed in both fixation methods that two miniplates or one reconstruction plates were used. In clinical settings, the use of a reconstruction metal plate has the disadvantage of causing small injuries on the fibular segment and affects the ability to use the same metal plates after flap failure. However, fixation using large metal plates can still lead to metal plate exposure, so that miniplates may be a better option [19]. Of the five patients in the current cases, three had their FFF fixed with miniplates. Although a definitive conclusion cannot be drawn based on the observations from the five patients, we may conclude based on previous findings and present observations that inexperienced surgeons must have a full understanding of the method of fixation before performing mandibular reconstruction using FFF and should consider the use of metal plates measuring 2.3 mm or greater.

During a mandibular reconstruction with FFF, it is important to determine the number of bone segments. When the number is too high, the blood flow into each bone segment decreases while injuries due to screw fixation increase. To reconstruct a large region of the mandible accurately, one must maintain the chin within a safe limit, increase contact between bone segments, and minimize defects [20]. Of the five patients in the current cases, two had their FFF bent once and fixed with a reconstruction metal plate while three patients had their FFF fixed with miniplates without bending. It appears unlikely that condylar dislocation occurred as a result of the large number of bone segments in this study. The panoramic radiographs obtained during the postoperative follow-up showed severe changes in the angle at the connection between the remaining mandibular bone and the FFF relative to the angle immediately after surgery. The mandibular condyle may have become displaced due to problems that arose during bone healing or fixation. However, the radiographs taken immediately after surgery showed satisfactory contact between the fibular bone segment and the remaining mandibular bone. The displacement thus appears to be the result of bone regeneration and jawbone functional recovery over a long period after surgery. Metal plates must be maintained for a long term after surgery. However, they can result in fatigue fracture and subsequent metal plate exposure [19]. Metal plate hardness and long-term fixation may not be the sole causes of metal plate exposure. A liquid diet to restrict a patient’s mandibular function may be considered.

To address issues regarding bone segment movement and condylar shape, CAD/CAM technology has been utilized to make customized FFF and condylar prostheses as well as to reconstruct the mandible [14, 21]. VSP and CAD/CAM technology help produce a reconstruction plate including the condyle that matches the shape and position of the resected anatomical structure. This accounts for fixation and condylar shape and position while also producing an FFF based on the patient’s mandibular shape. This reconstruction protocol may be expanded. Patients who underwent reconstruction using customized CAD/CAM plates that match the shape of the articular meniscus and cartilage, and who also received FFF, showed stable occlusion and mandibular function, as well as satisfactory recovery and esthetic outcomes. They had no joint pain around the TMJ or loosening of the metal plate. Resorption of the glenoid fossa did not occur, and the condylar region was displaced by 3.8 mm after surgery. A 5-year follow-up of patients who had undergone mandibular reconstruction under this protocol showed a shift of 0.19 mm, a 2.92 mm downward displacement of the condyle, and increased thickness of the glenoid fossa. Based on the observations of the five patients in the current cases, a mandibular reconstruction protocol which simultaneously uses CAD/CAM customized metal plates in the form of a condyle and FFF may be recommended.

It must be possible to produce a customized CAD/CAM metal plate in the form of a condyle by 3D printing based on VSP and CAD/CAM technologies. Although the technical requirements and production costs can be considered drawbacks, they are negligible considering how beneficial they are in overcoming the complications observed in the five patients in the current cases. A surgeon must thus have sufficient experience with CAD/CAM metal plates. Even if the surgeon is experienced in existing surgical methods, the use of VSP and CAD/CAM metal plates may help improve the surgical outcomes of patients. An inexperienced surgeon must use an RP model in addition to VSP and CAD/CAM metal plates to learn about the patient and the procedure before the actual surgery and to minimize as many errors as possible.

**Fig. 11:**
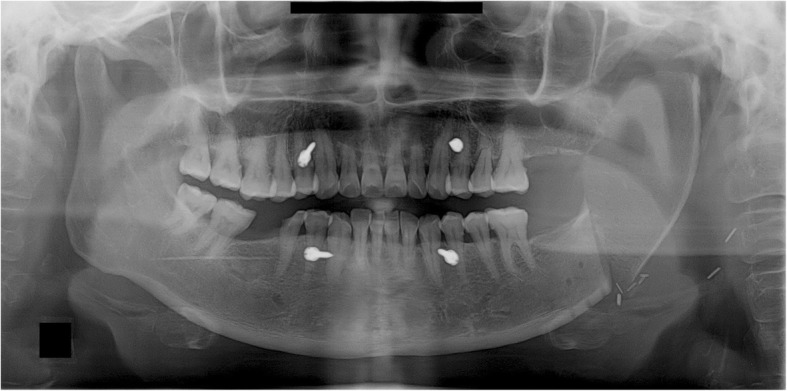
A panoramic radiograph obtained immediately after reconstruction using an iliac crest flap with deep circumplex iliac artery (DCIA) following left mandibular resection

**Fig. 12:**
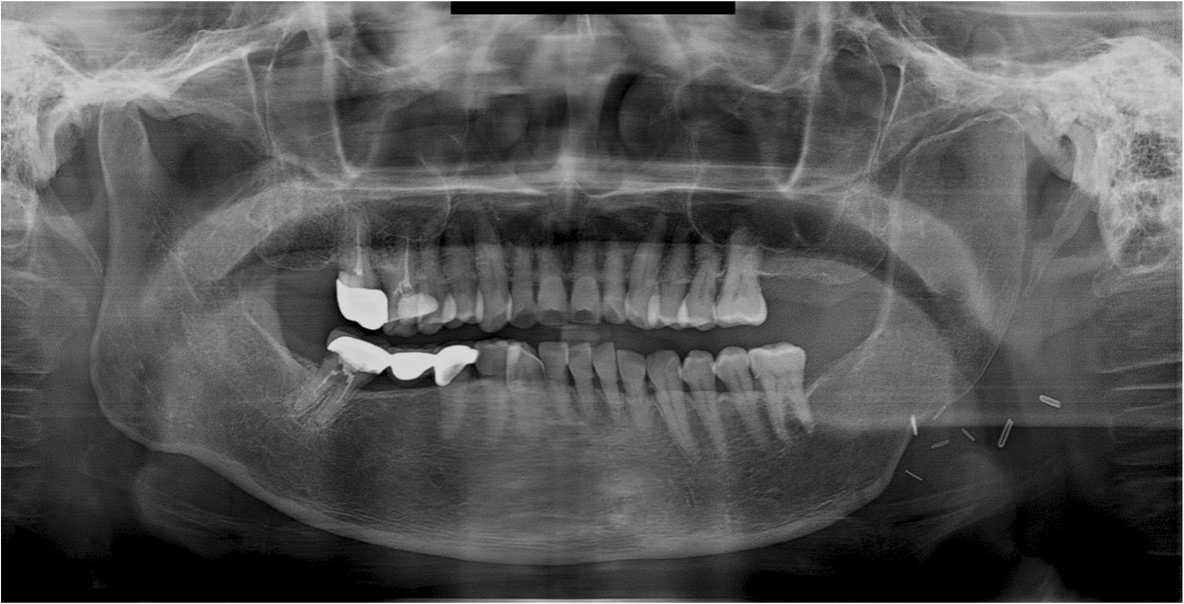
A panoramic radiograph obtained 10 years after reconstruction using DCIA flap following left mandibular resection

None of the five patients in the current cases experienced pain in the TMJ or severe trismus upon opening the mouth even when their condyles became severely displaced. Magnetic resonance imaging (MRI) was taken for the patient of case 1, 2, and 4 in the postoperative period. Postoperative MRI did not show the specific sign in the TMJ including of the glenoid fossa area. These complications of condylar displacement may be due to the gradual return to full mandibular function after reconstruction, positional and angular changes occurring slowly as healing took place between the bone segments. The force related to mandibular function consistently induces bone movements and causes metal plates to break and bend. This may occur when the flap height or thickness does not match that of the mandible. The deep circumflex iliac artery (DCIA) flap derived from the iliac bone has a thickness and shape similar to the mandibular bone and can be used in mandibular reconstruction. In DCIA reconstruction, the height of the bone segment was greater than that of the fibula, and the portion of the mandible from the angle to the mandibular condylar region could be reconstructed without bending the bone segments (Figs. 11 and 12). In this case, a stable connection can be achieved between the iliac bone and the mandibular bone remaining after mandibular resection. The DCIA flap may be considered in mandibular reconstruction involving the mandibular condyle [22, 23].

In addition, a scapular flap maintains a more bone stable contact than a fibula flap and enables a reconstruction that resembles the mandibular shape. However, using a scapular flap requires the patient’s position to be shifted during the reconstructive procedure. Moreover, two teams cannot perform mandibular resection and flap donor site acquirement at the same time [24].

## Conclusion

Although there are no clinical symptoms following a mandibular reconstruction using an FFF, various factors can cause severe displacement of the mandibular condyle away from the glenoid fossa after surgery. These cases had been performed by one surgeon. Although the exact complication rate cannot be calculated, authors recommend that surgeons must consider the condylar complication following mandibular reconstruction using FFF. This report of complication cases means the possibility of condylar displacement. A surgeon must have sufficient time to consider the use of a long flap with thickness similar to that of the mandible, ways to minimize span and bending, and methods of fixation. The patient, moreover, should be educated on condylar dislocation. Customized CAD/CAM-prototyped temporomandibular condyle-connected plates may be a good alternative even if VSP is to be performed before surgery. The DCIA flap may be considered in mandibular reconstruction involving the mandibular condyle. These considerations may help reduce the incidence of complications after mandibular reconstruction.

## Abbreviations

CAD/CAM: Computer-aided design/computer aided manufacturing
CT: Computed tomography
DCIA: Deep circumflex iliac artery
FFF: Fibula free flap
RP: Rapid prototyping
STL: Stereolithography
TMJ: Temporomandibular joint
VSP: Virtual surgical planning
